# Association of diabetes with coronary artery calcium in South Asian adults and other race/ethnic groups: The multi-ethnic study of atherosclerosis and the mediators of atherosclerosis in South Asians living in America study

**DOI:** 10.1177/14791641231204368

**Published:** 2023-10-05

**Authors:** Ned Premyodhin, Wenjun Fan, Millie Arora, Matthew J Budoff, Alka M Kanaya, Namratha Kandula, Latha Palaniappan, Jamal S Rana, Masood Younus, Nathan D Wong

**Affiliations:** 1 Division of Cardiology, 8788 University of California, Irvine, Irvine, CA, USA; 2 Department of Medicine, 8785 University of California, San Francisco, San Francisco, CA, USA; 3 Harbor-UCLA Medical Center, 117316 Lundquist Institute, Torrance, CA, USA; 4 Department of Medicine, Northwestern University, Chicago, IL, USA; 5 Department of Medicine, Stanford University, Palo Alto, CA, USA; 6 Department of Cardiology, 6152 Kaiser Permanente, Oakland, CA, USA; 7 Department of Medicine, 8789 University of California, Davis, Sacramento, CA, USA

**Keywords:** Coronary artery calcium, atherosclerosis, diabetes mellitus, race, ethnicity

## Abstract

**Purpose:**

South Asian (SA) persons have increased risks for diabetes mellitus (DM) and atherosclerotic cardiovascular disease (ASCVD). We examined whether the association of DM with subclinical atherosclerosis assessed by coronary artery calcium (CAC) differs in SA versus other ethnic groups.

**Methods:**

We studied adults from the Multi-Ethnic Study of Atherosclerosis and the Mediators of Atherosclerosis in South Asians Living in America studies without ASCVD. CAC was examined among those normoglycemic, pre-DM and DM. Logistic regression examined pre-DM and DM with the odds of any CAC > 0 and CAC ≥ 100.

**Results:**

Among 7562 participants, CAC > 0 and CAC ≥ 100 in those with DM was highest in non-Hispanic White (NHW) (80% and 48%) and SA (72% and 41%) persons. Adjusted Ln (CAC + 1) was highest in NHW (3.68 ± 0.21) and SA (3.60 ± 0.23) (*p* < .01) DM patients. SA and NHW adults with DM (vs normoglycemic) had highest odds of CAC > 0 (2.13 and 2.27, respectively, *p* < .01). For CAC ≥ 100, SA and Chinese adults had the highest odds (2.28 and 2.27, respectively, *p* < .01). Fasting glucose and glycated hemoglobin were most strongly associated with CAC among SA.

**Conclusions:**

Diabetes mellitus most strongly relates to any CAC in SA and NHW adults and CAC ≥ 100 in SA and Chinese adults, helping to explain the relation of DM with ASCVD in these populations.

## Introduction

South Asian (SA) adults have greater risks for atherosclerotic cardiovascular disease (ASCVD) and diabetes mellitus (DM) with greater morbidity and mortality compared to other racial and ethnic groups that is incompletely attributable to differences in traditional risk factors.^1–3^ The prevalence and extent of coronary artery calcium (CAC) as a measure of subclinical atherosclerosis burden in SA adults compared to adults of other race/ethnic groups has been explored.^4^ Previously, a higher CAC burden and a greater prevalence of multi-vessel disease and involvement of the left anterior descending coronary artery has also been noted in SA adults with diabetes mellitus (DM) compared to non-Hispanic White adults (NHW) with DM.^5^ However, the relation of glycemic status with CAC burden in SA adults compared to NHW adults and adults of other race/ethnic groups has not been evaluated in large population-based studies. As DM may be as much as 3 times more prevalent in SA adults compared to NHW adults,^2^ this association may have implications for more severe clinical ASCVD observed in SA individuals. Furthermore, as the SA population is a rapidly growing minority group in the United States and other Western countries, reduction of ASCVD risk in this population is a clinical and public health imperative. The Multi-Ethnic Study of Atherosclerosis (MESA) and Mediators of Atherosclerosis in South Asians Living in America (MASALA) study are prospective studies investigating the prevalence, risk factors, and outcomes of ASCVD in NHW, Chinese (CH), African American (AA) and Hispanic (HS) adults (MESA)^6^ and SA adults (MASALA).^7^ We used data from these studies to explore the importance of glycemic status as an indicator of any, and significant CAC and how this relation varies by race/ethnicity.

## Patients and methods

### Study design

This study was designed to explore the role of DM on ASCVD trends between self-identified race/ethnicity groups using the MESA and MASALA studies. Self-identified race/ethnicity was not considered a risk factor, nor a proxy for any other risk factors which are adjusted for in our analyses. No assumptions were made regarding self-identified race/ethnicity and its effect on prevalence of CAC or ASCVD. The rationale, design, and methods of the MESA and MASALA studies have previously been published.^6,7^ Both studies are prospective, community-based studies of individuals with no prior history of cardiovascular disease with measures of risk factors. The inclusion criteria for participation in both studies were nearly identical with those of MESA found online at https://www.mesa-nhlbi.org/. In brief, exclusion criteria included physician-diagnosed myocardial infarction, heart failure, stroke or transient ischemic attack, angina or the use of nitroglycerin, current atrial fibrillation, or history of having undergone any procedures related to cardiovascular disease or any surgery on the heart or arteries. Pregnant individuals or those receiving active cancer treatment were also excluded. Due to limitations of imaging systems for computed tomography (CT) scanning, participants weighing greater than 300 lbs were excluded. Both studies utilized similar telephone-based recruitment methods in conjunction with brochures and community outreach. MESA included 6814 men and women, aged 45–84 years with no prior history of ASCVD at baseline. Participants self-identified as NHW, CH, AA or HS adults and were recruited from six communities in the United States: Baltimore County, Maryland; Greater Chicago, Illinois; Forsyth County, North Carolina; Los Angeles County, California; New York, New York; and St. Paul, Minnesota. Enrollment occurred between July 2000 and July 2002. Participants provided written informed consent upon arrival to enrollment centers, and Institutional Review Boards at respective centers approved the study.

The MASALA study was developed using methods and measures from the MESA study to enable direct comparison of the adult SA population in the United States to the MESA participants, by race and ethnicity. MASALA included 906 individuals enrolled from 2 clinical sites in the United States: The University of California, San Francisco and Northwestern University in Chicago, Illinois. Participants were enrolled from October 2010 to March 2013. Enrollees were stratified by sex and age to ensure an equal distribution for each age decade from ages 40–84. To be eligible, individuals must have had 3 or more grandparents born in Bangladesh, India, Nepal, Pakistan, or Sri Lanka. Participants provided written informed consent. MASALA was approved by the Institutional Review Boards of the University of California, San Francisco, and Northwestern University.

### Study population and measures

For the present study, participants enrolled in the first examination of both MESA and MASALA were combined and the variables of interest were recoded as needed for consistency in analysis. Individuals aged <45 years were excluded from MASALA to match the MESA participant age range. Participants who had CAC data, DM status, and medical history were included.

Identical questionnaires were administered to participants of both studies to collect harmonized demographic and socioeconomic data (including education and access to care), language information, social history including alcohol and tobacco use, family history of cardiovascular disease, and prescription medication, non-prescription medication and supplement use.

Height, weight, waist and hip circumferences were measured. Resting blood pressure was measured 3 times in the seated position using automated sphygmomanometers and the average of the last two measurements was used for analysis. Hypertension was defined as use of any antihypertensive medication or a systolic blood pressure (SBP) ≥ 140 mmHg or diastolic blood pressure (DBP) ≥ 90 mmHg, consistent with guidelines at the time of study enrollment. Taking a medication from any one of the following classes qualified as “use of any lipid lowering medication”: statins, fibrates, or niacin.^4^

Diabetes mellitus status was defined in both MESA and MASALA following the 2003 American Diabetes Association criteria.^8^ In both studies, fasting plasma glucose was measured for all participants. Impaired fasting glucose or pre-diabetes (pre-DM) was defined as fasting glucose of 100–125 mg/dL (5.6 – 6.9 mmol/L). DM was defined as fasting plasma glucose ≥ 126 mg/dL (≥7.0 mmol/L) or taking insulin or oral diabetes medication. DM was additionally stratified by duration of DM (≥10 years vs <10 years). Total cholesterol, triglycerides (TG), and high-density lipoprotein cholesterol (HDL-C) were measured using industry standard enzymatic assays while low-density lipoprotein cholesterol (LDL-C) was calculated.^4,6,7^ Estimated glomerular filtration rate (eGFR) was estimated using the CKD-EPI equation as previously described.^9^

In MASALA, CAC was measured from non-contrast cardiac CT scans obtained using a cardiac-gated electron-beam CT scanner at UCSF (16D scanner, Philips Medical Systems, Andover, MA) or the MSD Aquilion 64 model (Toshiba Medical Systems, Tustin, CA). At Northwestern University, the Sensation Cardiac 64 Scanner (Siemens Medical Solutions, Malvern, PA) was used. Participants were scanned in the supine position with both arms raised above the head and oriented traditionally by CT standards. The scanning and resolution settings, exposure voltage and currents per unit weight and image reconstruction protocols were previously published.^7^ In MESA, CAC was measured using either a cardiac-gated electron-beam CT scanner (Imatron C-150; Imatron, South San Francisco, California) or a prospectively electrocardiogram-triggered scan acquisition at 50% of the R-R interval with a multidetector CT system depending on the site.^6^ All CT scans for both studies were read at Harbor-UCLA Medical Center (Torrance, CA) in accordance with MESA study methods. Values were reported as phantom adjusted Agatston calcium scores and total calcium volumes. Clinically significant CAC was defined as a score of ≥100, given this level has been recommended in guidelines for treatment.^10^

### Statistical analysis

For baseline characteristics, participants were classified by race/ethnicity and according to glycemic status: normal fasting glucose, pre-DM, or DM. Multiple group comparisons across race/ethnic groups within each DM status were performed using analysis of variance for continuous variables and the Chi-squared test of proportions for categorical variables. Participants were stratified into CAC categories by conventional CAC score ranges: 0, 1–99, 100–399, and ≥ 400.^11^ The distribution of CAC was described in terms of percentage of individuals within each CAC score for each race/ethnic group and by DM status. CAC values were also analyzed as continuous variables after normalization by calculating the natural log (ln) transformation of the phantom adjusted CAC score +1, or Ln (CAC + 1).^11^

Analysis of covariance (ANCOVA) was used to analyze CAC burden for participants with pre-DM, DM, or neither condition by calculating the least squared means of Ln (CAC + 1) for each race/ethnic group within each diabetes category. Two models were created, an unadjusted model and a model adjusting for all relevant covariates including age, sex, family history, education (attainment of bachelor’s degree or higher), smoking status, SBP, DBP, LDL-C, HDL-C, TG, eGFR, waist circumference, activity level, use of lipid lowering medication, and use of antihypertensive medication with *p* < .05 considered to be significant.

Multiple logistic regression calculated the odds ratios (ORs) for having any CAC (score > 0) and clinically significant CAC (defined as CAC ≥ 100) after adjusting for the same covariates as in the ANCOVA analysis for those with pre-DM and DM compared to those with neither condition. For continuous variables, ORs were expressed per standard deviation increment, with the exception of age for which an increase of 10 years was used. The interaction of DM status and race/ethnicity was also tested using multiple logistic regression for both any CAC > 0 and clinically significant CAC ≥ 100. A linear regression analysis was performed to examine and compare the slope of Ln (CAC + 1) with both fasting glucose and HbA1c across race/ethnicity in persons with CAC > 0. The interaction of fasting glucose and HbA1c by race/ethnicity with Ln (CAC + 1) was also tested. All statistical analysis was performed using Statistical Analysis Software (SAS) version 9.4 (SAS Institute, Cary, NC).

## Results

We included a total of 7562 participants in this analysis, of whom 5405 (71.5%) had normal fasting glucose, 1131 (15.0%) had pre-DM, and 1026 (13.6%) had DM (Table 1). Of those without DM, the majority were NHW adults (40%) followed by AA adults (23.5%). The average age of persons with DM was slightly higher compared to those without DM for all race/ethnic groups. SA adults from MASALA were slightly younger on average than MESA participants. With the exception of AA adults with DM (50.9% female), the pre-DM and DM groups had a greater proportion of men than women among all subgroups. Conversely, those without DM had a greater percentage of women than men for all groups, including SA adults (54.4% female). Average waist circumference, fasting glucose, SBP and percentage of participants using lipid lowering medications was higher across DM categories for all race or ethnic groups while a less consistent trend was observed for smoking status, LDL-C, TG, and HDL-C. The percentage of participants with a bachelor’s degree or higher was lower with pre-DM and DM compared to non-DM.

**Table 1.** Baseline characteristics by race/ethnic group and glucose group, MASALA and MESA.

	No diabetes					Pre-diabetes					Diabetes				
	Non-Hispanic White	Chinese	African American	Hispanic	South Asian	Non-Hispanic White	Chinese	African American	Hispanic	South Asian	Non-Hispanic White	Chinese	African American	Hispanic	South Asian
	*N* = 2160 (40.0%)	*N* = 558 (10.3%)	*N* = 1271 (23.5%)	*N* = 997 (18.5%)	*N* = 419 (7.8%)	*N* = 294 (26.0%)	*N* = 137 (12.1%)	*N* = 277 (24.5%)	*N* = 231 (20.4%)	*N* = 192 (17.0%)	*N* = 158 (15.4%)	*N* = 105 (10.2%)	*N* = 332 (32.4%)	*N* = 264 (25.7%)	*N* = 167 (16.3%)
Age (years)	62.1 ± 10.3	61.3 ± 10.3	61.2 ± 10.2	59.9 ± 10.3	56.6 ± 8.3	64.9 ± 9.8	64.1 ± 10.3	63.9 ± 9.6	63.9 ± 9.9	57.3 ± 8.2	65.3 ± 9.7	66.2 ± 9.2	64.4 ± 9.1	64.2 ± 9.7	59.6 ± 8.1
Sex															
Male (%)	990 (45.8)	251 (45.0)	529 (41.6)	457 (45.9)	191 (45.6)	172 (58.5)	82 (59.9)	145 (52.4)	126 (54.6)	113 (58.9)	95 (60.1)	54 (51.4)	163 (49.1)	137 (51.9)	112 (67.1)
Female (%)	1172 (54.2)	307 (55.0)	742 (58.4)	540 (54.2)	228 (54.4)	122 (41.5)	55 (40.2)	132 (47.7)	105 (45.5)	79 (42.2)	63 (39.9)	51 (48.6)	169 (50.9)	127 (48.1)	55 (32.9)
BMI (kg/m^2^)	27.2 ± 4.8	23.6 ± 3.2	29.4 ± 5.8	28.6 ± 4.6	25.3 ± 3.7	30.1 ± 5.2	25.0 ± 3.5	31.9 ± 5.9	31.1 ± 5.1	26.7 ± 4.2	31.2 ± 5.5	24.8 ± 3.1	31.5 ± 5.6	31.2 ± 5.8	26.5 ± 4.3
Waist circumference (cm)	96 ± 14	86 ± 10	99 ± 14	98 ± 13	91 ± 10	105 ± 13	90 ± 9	106 ± 15	106 ± 12	95 ± 10	109 ± 14	91 ± 9	107 ± 14	106 ± 14	96 ± 11
Activity (MET-min/week)	4226 (2340–7140)	2683 (1410–5347)	4683 (2250–8788	4642 (1966–8430)	9592 (7455–11910)	4395 (1995–7515)	2655 (1335–4530)	4320 (1987–7995)	3645 (1605–7320)	9266 (7267–12345)	3787 (2122–7080)	2362 (1050–3967)	3825 (1800–7860)	2865 (1080–7803)	8857 (6785–11348)
Smoking (%)															
Never	957 (44.4)	431 (77.0)	565 (44.8)	560 (56.1)	360 (85.9)	130 (44.4)	98 (72.1)	128 (46.7)	112 (48.5)	153 (79.7)	68 (43.0)	74 (46.8)	149 (45.2)	134 (50.8)	128 (76.7)
Prior	953 (44.2)	101 (18.0)	454 (36.0)	300 (30.0)	45 (10.7)	126 (43.0)	30 (22.1)	110 (40.2)	87 (37.7)	33 (17.2)	74 (46.8)	22 (21.0)	127 (38.5)	98 (37.1)	32 (19.2)
Current	246 (11.4)	28 (5.0)	243 (19.3)	139 (13.9)	14 (3.3)	37 (12.6)	8 (5.9)	36 (13.1)	32 (13.9)	6 (3.1)	16 (10.1)	9 (8.6)	54 (16.4)	32 (12.1)	7 (4.2)
Use of lipid lowering med (%)	361 (16.7)	54 (9.6)	165 (13.0)	107 (10.7)	79 (18.9)	70 (23.9)	26 (19.0)	48 (17.5)	30 (13.0)	63 (32.8)	50 (31.7)	36 (34.3)	98 (29.7)	58 (22.1)	104 (62.3)
Family history of MI (%)	1030 (50.5)	110 (21.2)	495 (41.5)	375 (39.6)	195 (46.7)	153 (55.6)	21 (17.2)	118 (45.9)	85 (39.4)	85 (44.5)	85 (57.8)	16 (17.0)	129 (40.6)	105 (44.1)	87 (52.1)
Bachelors degree (%)	1113 (51.5)	227 (40.7)	462 (36.3)	117 (11.7)	369 (88.1)	120 (40.8)	48 (35.0)	82 (29.6)	18 (7.8)	165 (85.9)	59 (37.3)	34 (32.4)	89 (26.8)	13 (4.9)	142 (85.0)
SBP (mmHg)	122.1 ± 20.0	122.6 ± 21.2	129.8 ± 21.4	123.8 ± 21.5	123.7 ± 15.8	130.5 ± 20.9	128.5 ± 22.7	135.1 ± 21.4	132.3 ± 19.9	127.9 ± 16.3	129.1 ± 21.4	130.1 ± 20.3	136.1 ± 21.5	132.9 ± 23.0	130.1 ± 14.6
DBP (mmHg)	69.9 ± 9.8	71.6 ± 10.0	74.2 ± 10.2	71.2 ± 10.1	72.2 ± 10.1	72.6 ± 10.7	73.6 ± 11.4	76.1 ± 10.4	74.4 ± 10.0	75.2 ± 10.1	69.7 ± 9.7	71.8 ± 10.0	74.3 ± 10.3	70.6 ± 10.1	74.5 ± 8.8
Total cholesterol (mg/dL) [nmol/L]	196.1 ± 34.2 [5.1 ± 0.9]	193.0 ± 31.8 [5.0 ± 0.8]	190.9 ± 36.2 [4.9 ± 0.9]	199.0 ± 36.3 [5.1 ± 0.9]	192.5 ± 34.2 [5.0 ± 0.9]	196.7 ± 36.0 [5.1 ± 0.9]	195.0 ± 31.8 [5.0 ± 0.8]	187.5 ± 35.4 [4.8 ± 0.9]	199.8 ± 35.2 [5.2 ± 0.9]	189.6 ± 34.7 [4.9 ± 0.9]	188.3 ± 44.1 [4.9 ± 1.1]	186.7 ± 30.8 [4.8 ± 0.8]	186.5 ± 37.4 [4.8 ± 1.0]	192.3 ± 43.1 [5.0 ± 1.1]	170.4 ± 41.5 [4.4 ± 1.1]
LDL-C (mg/dL) [nmol/L]	117.5 ± 30.0 [3.0 ± 0.8]	115.5 ± 29.1 [3.0 ± 0.8]	117.0 ± 32.9 [3.0 ± 0.9]	121.1 ± 31.6 [3.1 ± 0.8]	115.4 ± 30.8 [3.0 ± 0.8]	118.3 ± 30.2 [3.1 ± 0.8]	117.6 ± 28.6 [3.0 ± 0.7]	116.5 ± 32.9 [3.0 ± 0.9]	121.0 ± 31.0 [3.1 ± 0.8]	113.0 ± 30.0 [2.9 ± 0.8]	108.0 ± 30.1 [2.8 ± 0.8]	108.3 ± 27.2 [2.8 ± 0.7]	114.5 ± 33.5 [2.9 ± 0.9]	112.3 ± 37.9 [2.9 ± 1.0]	94.3 ± 33.1 [2.4 ± 0.9]
HDL-C (mg/dL) [nmol/L]	53.4 ± 15.9 [1.4 ± 0.4]	50.8 ± 12.7 [1.3 ± 0.3]	54,3 ± 15.9 [1.4 ± 0.4]	49.3 ± 13.5 [1.3 ± 0.3]	52.3 ± 14.2 [1.4 ± 0.4]	47.5 ± 13.2 [1.2 ± 0.3]	48.1 ± 12.5 [1.2 ± 0.3]	49.2 ± 13.0 [1.3 ± 0.3]	44.0 ± 11.1 [1.1 ± 0.3]	48.9 ± 12.4 [1.3 ± 0.3]	45.3 ± 13.8 [1.2 ± 0.4]	44.7 ± 11.6 [1.2 ± 0.3]	47.8 ± 13.0 [1.2 ± 0.3]	44.5 ± 11.7 [1.2 ± 0.3]	47.0 ± 11.5 [1.2 ± 0.3]
Triglycerides (mg/dL) [nmol/L]	126.1 ± 73.2 [1.4 ± 0.8]	134.1 ± 72.8 [1.5 ± 0.8]	97.6 ± 54.6 [1.1 ± 0.6]	143.6 ± 86.9 [1.6 ± 1.0]	124.3 ± 69.2 [1.4 ± 0.8]	154.3 ± 94.6 [1.7 ± 1.1]	148.8 ± 85.3 [1.7 ± 1.0]	109.2 ± 53.8 [1.2 ± 0.6]	182.7 ± 109.5 [2.1 ± 1.2]	138.3 ± 62.6 [1.6 ± 0.7]	185.2 ± 201.7 [2.1 ± 2.3]	180.2 ± 124.7 [2.0 ± 1.4]	128.7 ± 110.2 [1.5 ± 1.2]	185.5 ± 129.9 [2.1 ± 1.5]	145.1 ± 85.1 [1.6 ± 1.0]
Fasting glucose (mg/dL) [nmol/L]	92.2 ± 7.4 [5.1 ± 0.4]	94.7 ± 6.3 [5.3 ± 0.3]	93.4 ± 7.1 [5.2 ± 0.4]	93.9 ± 7.1 [5.2 ± 0.4]	90.4 ± 5.7 [5.0 ± 0.3]	114.6 ± 6.8 [6.4 ± 0.4]	114.2 ± 7.2 [6.3 ± 0.4]	114.9 ± 7.4 [6.4 ± 0.4]	115.2 ± 7.2 [6.4 ± 0.4]	107.1 ± 6.5 [5.9 ± 0.4]	153.0 ± 56.3 [8.5 ± 3.1]	156.0 ± 52.8 [8.7 ± 2.9]	153.6 ± 53.6 [8.5 ± 3.0]	171.1 ± 63.5 [9.5 ± 3.5]	137.8 ± 37.0 [7.7 ± 2.1]
Glycated hemoglobin (%)	5.3 ± 0.4	5.5 ± 0.4	5.5 ± 0.4	5.4 ± 0.4	5.7 ± 0.3	5.8 ± 0.7	5.9 ± 0.8	6.1 ± 0.8	6.0 ± 0.7	5.9 ± 0.4	6.9 ± 1.3	7.3 ± 1.4	7.4 ± 1.8	7.8 ± 1.8	7.3 ± 1.3
eGFR (mL/min/1.73 m^2^)	73.7 ± 14.0	80.4 ± 15.3	80.5 ± 16.9	80.2 ± 14.9	91.3 ± 12.7	74.2 ± 14.6	76.3 ± 15.2	79.4 ± 18.1	79.2 ± 15.7	89.6 ± 12.7	74.8 ± 16.8	79.3 ± 17.1	80.6 ± 21.8	81.2 ± 20.7	87.5 ± 16.5
Total cholesterol (mg/dL) [nmol/L]	196.1 ± 34.2 [5.1 ± 0.9]	193.0 ± 31.8 [5.0 ± 0.8]	190.9 ± 36.2 [4.9 ± 0.9]	199.0 ± 36.3 [5.1 ± 0.9]	192.5 ± 34.2 [5.0 ± 0.9]	196.7 ± 36.0 [5.1 ± 0.9]	195.0 ± 31.8 [5.0 ± 0.8]	187.5 ± 35.4 [4.8 ± 0.9]	199.8 ± 35.2 [5.2 ± 0.9]	189.6 ± 34.7 [4.9 ± 0.9]	188.3 ± 44.1 [4.9 ± 1.1]	186.7 ± 30.8 [4.8 ± 0.8]	186.5 ± 37.4 [4.8 ± 1.0]	192.3 ± 43.1 [5.0 ± 1.1]	170.4 ± 41.5 [4.4 ± 1.1]
LDL-C (mg/dL) [nmol/L]	117.5 ± 30.0 [3.0 ± 0.8]	115.5 ± 29.1 [3.0 ± 0.8]	117.0 ± 32.9 [3.0 ± 0.9]	121.1 ± 31.6 [3.1 ± 0.8]	115.4 ± 30.8 [3.0 ± 0.8]	118.3 ± 30.2 [3.1 ± 0.8]	117.6 ± 28.6 [3.0 ± 0.7]	116.5 ± 32.9 [3.0 ± 0.9]	121.0 ± 31.0 [3.1 ± 0.8]	113.0 ± 30.0 [2.9 ± 0.8]	108.0 ± 30.1 [2.8 ± 0.8]	108.3 ± 27.2 [2.8 ± 0.7]	114.5 ± 33.5 [2.9 ± 0.9]	112.3 ± 37.9 [2.9 ± 1.0]	94.3 ± 33.1 [2.4 ± 0.9]
HDL-C (mg/dL) [nmol/L]	53.4 ± 15.9 [1.4 ± 0.4]	50.8 ± 12.7 [1.3 ± 0.3]	54,3 ± 15.9 [1.4 ± 0.4]	49.3 ± 13.5 [1.3 ± 0.3]	52.3 ± 14.2 [1.4 ± 0.4]	47.5 ± 13.2 [1.2 ± 0.3]	48.1 ± 12.5 [1.2 ± 0.3]	49.2 ± 13.0 [1.3 ± 0.3]	44.0 ± 11.1 [1.1 ± 0.3]	48.9 ± 12.4 [1.3 ± 0.3]	45.3 ± 13.8 [1.2 ± 0.4]	44.7 ± 11.6 [1.2 ± 0.3]	47.8 ± 13.0 [1.2 ± 0.3]	44.5 ± 11.7 [1.2 ± 0.3]	47.0 ± 11.5 [1.2 ± 0.3]
Triglycerides (mg/dL) [nmol/L]	126.1 ± 73.2 [1.4 ± 0.8]	134.1 ± 72.8 [1.5 ± 0.8]	97.6 ± 54.6 [1.1 ± 0.6]	143.6 ± 86.9 [1.6 ± 1.0]	124.3 ± 69.2 [1.4 ± 0.8]	154.3 ± 94.6 [1.7 ± 1.1]	148.8 ± 85.3 [1.7 ± 1.0]	109.2 ± 53.8 [1.2 ± 0.6]	182.7 ± 109.5 [2.1 ± 1.2]	138.3 ± 62.6 [1.6 ± 0.7]	185.2 ± 201.7 [2.1 ± 2.3]	180.2 ± 124.7 [2.0 ± 1.4]	128.7 ± 110.2 [1.5 ± 1.2]	185.5 ± 129.9 [2.1 ± 1.5]	145.1 ± 85.1 [1.6 ± 1.0]
Mean adjusted CAC score	161.0 ± 411.3	58.4 ± 158.6	101.6 ± 365.6	94.7 ± 335.3	85.3 ± 318.0	324.7 ± 664.3	113.3 ± 246.1	114.4 ± 339.1	105.9 ± 270.3	146.9 ± 460.1	335.1 ± 586.2	266.6 ± 549.0	206.8 ± 560.7	265.1 ± 659.3	214.9 ± 440.5
Mean adjusted CAC volume	140.9 ± 345.2	53.0 ± 137.7	88.9 ± 304.0	84.6 ± 280.4	76.0 ± 267.0	279.1 ± 542.0	102.5 ± 210.3	101.0 ± 285.9	96.2 ± 225.9	129.7 ± 378.6	291.9 ± 485.7	234.9 ± 467.3	185.6 ± 478.4	235.0 ± 554.7	191.2 ± 367.0
Mean Ln (CAC + 1)	2.4 ± 2.6	1.8 ± 2.2	1.7 ± 2.3	1.7 ± 2.3	1.5 ± 2.3	3.4 ± 2.8	2.5 ± 2.4	1.9 ± 2.4	2.2 ± 2.4	2.0 ± 2.5	4.0 ± 2.5	3.4 ± 2.7	2.6 ± 2.7	2.8 ± 2.7	3.4 ± 2.5

Ethnicity percentages represent composition within each glucose group. All race/ethnic comparisons *p* < .01 except fasting glucose in the pre-diabetes group (*p* < .012), fasting glucose in the diabetes group (*p* = .723), LDL-C in the pre-diabetes group (*p* = .154), mean adjusted CAC score in the diabetes group (*p* = .183) and mean adjusted CAC volume in the diabetes group (*p* = .192). Abbreviations: BMI = body mass index, CAC = coronary artery calcium, DBP = diastolic blood pressure, HDL-C = high-density lipoprotein cholesterol, LDL-C = low-density lipoprotein cholesterol, MI = myocardial infarction, SBP = systolic blood pressure, eGFR = estimated glomerular filtration rate.

**Figure 1.**
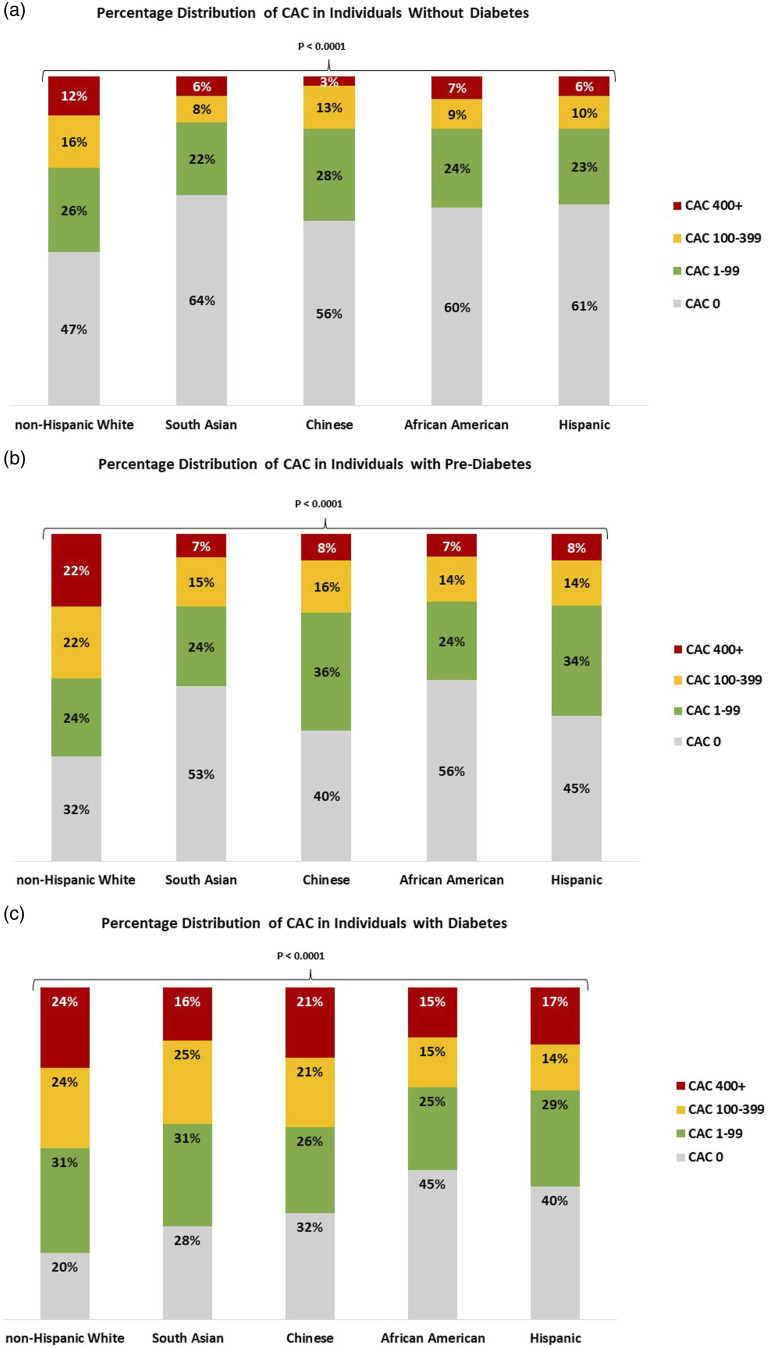
Unadjusted prevalence of coronary artery calcium by race/ethnic group, MESA and MASALA. Individual panels represent individuals with (a) Normal fasting glucose, (b) Pre-Diabetes and (c) Diabetes. Data are unadjusted for covariates. *p*-values represent cross ethnic comparisons within a glucose group. Abbreviations: CAC = coronary artery calcium.

Mean CAC score, mean phantom adjusted CAC volume and Ln (CAC + 1) were noted to be significantly higher in those with DM versus without DM for all groups (Table 1). The prevalence of CAC scores ≥ 100 was greater for pre-DM and DM adults compared to non-DM adults for both men and women (Figure 1). In individuals without DM, NHW had the highest prevalence of CAC scores ≥ 100 (range: 14% in SA adults to 28% in NHW adults). The prevalence of CAC ≥ 100 in those with pre-DM ranged from 21% in AA adults to 44% in NHW adults and for those with DM ranged from 30% in AA adults to 48% in NHW adults (*p* < .0001 across race or ethnicity for each DM group).

The unadjusted and adjusted Ln (CAC + 1) measures are shown in Table 2. Ln (CAC + 1) after adjusting for all covariates was highest in NHW adults followed by SA and CH adults and was lowest in AA adults for all glucose groups. In the DM group, NHW adults continued to exhibit the highest Ln (CAC + 1) of 3.68 ± 0.21, followed closely by SA adults at 3.60 ± 0.23, then CH adults at 3.46 ± 0.27 after adjusting for all covariates (Table 2). Moreover, when DM was stratified by duration, Ln (CAC + 1) was consistently higher in all race/ethnic groups for those with duration ≥10 years compared to <10 years.

**Table 2.** Values of Ln (CAC + 1) adjusted and unadjusted for risk factors for all glucose groups, MASALA and MESA.

	Unadjusted Ln (CAC + 1)						Adjusted Ln (CAC + 1) for all covariates					
	Non-Hispanic White	Chinese	African American	Hispanic	South Asian	*p*-value	Non-Hispanic White	Chinese	African American	Hispanic	South Asian	*p*-value
No diabetes	2.41 ± 0.05	1.75 ± 0.10	1.66 ± 0.07	1.65 ± 0.08	1.53 ± 0.12	*p* < .01	2.21 ± 0.05	2.04 ± 0.09	1.52 ± 0.06	1.74 ± 0.07	2.00 ± 0.11	*p* < .01
Pre-diabetes	3.39 ± 0.15	2.49 ± 0.21	1.93 ± 0.15	2.21 ± 0.17	2.04 ± 0.18	*p* < .01	2.95 ± 0.14	2.86 ± 0.21	1.62 ± 0.14	2.05 ± 0.16	2.82 ± 0.19	*p* < .01
Diabetes	3.97 ± 0.21	3.37 ± 0.26	2.60 ± 0.14	2.81 ± 0.16	3.42 ± 0.20	*p* < .01	3.68 ± 0.21	3.46 ± 0.27	2.68 ± 0.14	2.90 ± 0.17	3.60 ± 0.23	*p* < .01
DM < 10 years	3.96 ± 0.23	3.05 ± 0.28	2.41 ± 0.17	2.42 ± 0.19	3.04 ± 0.26	*p* < .01	3.51 ± 0.23	3.13 ± 0.29	2.56 ± 0.17	2.57 ± 0.20	3.27 ± 0.28	*p* < .01
DM> = 10 years	3.99 ± 0.46	4.93 ± 0.63	3.06 ± 0.27	3.79 ± 0.31	4.00 ± 0.33	*p* = .03	4.15 ± 0.47	4.73 ± 0.67	2.90 ± 0.30	3.71 ± 0.34	4.33 ± 0.42	*p* < .01

*p*-values were calculated across all racial/ethnic groups within a glucose group. Data shown includes an unadjusted model and a model adjusting for all relevant risk factors including age, sex, family history, education (attainment of bachelor’s degree or higher), smoking status, systolic blood pressure, diastolic blood pressure, low-density lipoprotein cholesterol, high-density lipoprotein cholesterol, triglyceride level, eGFR, waist circumference, physical activity level, use of lipid lowering medication and use of antihypertensive medication with *p* < .05 considered to be significant.

From fully adjusted multiple logistic regression, compared to normoglycemia, pre-DM was not associated with higher odds of having any CAC (CAC score > 0) or clinically significant CAC (CAC score ≥ 100) (Table 3). Having DM was associated with significantly higher odds of having any CAC for NHW and SA persons, but not for CH, AA or HS persons. Having DM was associated with the highest odds of having a clinically significant CAC score ≥ 100 in CH adults (OR 2.27, *p* < .01) and SA adults (OR 2.28, *p* < .01). NHW and AA adults with DM demonstrated lower though still significant odds of having clinically significant CAC compared to not having DM (ORs 1.56, *p* < .05 and 1.62, *p* < .01, respectively). The interaction of DM status and race/ethnicity was not significant for any CAC (*p* = .0647) but was significant for CAC score ≥ 100 (*p* = .0239) (Table 3). Importantly, when stratifying by DM duration, the odds of any CAC and significant CAC were highest for those with duration ≥10 years, being 3.64 (*p* < .05) and 3.05 (*p* < .01) for any CAC among NHW and SA persons, respectively. For clinically significant CAC ≥ 100, these odds were 3.75, 5.81, and 2.74 among NHW, CH, and SA adults, respectively (all *p* < .01), with an interaction *p* = .05.

**Table 3.** Adjusted odds ratios of having any CAC (score > 0) or clinically significant CAC (score ≥ 100) for pre-diabetes and diabetes compared to neither condition.

	Any CAC (score > 0)					Clinically significant CAC (score ≥ 100)				
	non-Hispanic White	Chinese	African American	Hispanic	South Asian	non-Hispanic White	Chinese	African American	Hispanic	South Asian
No diabetes	Reference	Reference	Reference	Reference	Reference	Reference	Reference	Reference	Reference	Reference
Pre-diabetes	1.02 (0.73 - 1.41)	1.33 (0.84 - 2.13)	0.81 (0.59 - 1.12)	1.09 (0.76 - 1.58)	1.15 (0.75 – 1.77)	1.37 (0.99 – 1.89)	1.17 (0.67 – 2.03)	0.88 (0.59 - 1.30)	0.78 (0.50 - 1.24)	1.29 (0.76 - 2.17)
Diabetes	2.27** (1.38 - 3.74)	1.41 (0.82 - 2.43)	1.34 (0.99 - 1.81)	1.40 (0.96 – 2.06)	2.13** (1.28 – 3.55)	1.56* (1.02 - 2.39)	2.27** (1.29 - 3.98)	1.62** (1.15 - 2.27)	1.49 (0.98 - 2.26)	2.28** (1.35 - 3.87)
DM < 10 years	1.94* (1.11-3.39)	1.36 (0.76-2.44)	1.30 (0.92-1.82)	1.28 (0.83-1.96)	1.96* (1.07-3.57)	1.26 (0.78-2.01)	2.17* (1.15-4.07)	1.50* (1.01-2.21)	1.37 (0.84-2.23)	2.12* (1.14-3.93)
DM ≥ 10 years	3.64* (1.32-10.06)	2.95 (0.60-14.51)	1.38 (0.83-2.31)	1.96 (0.97-3.97)	3.05** (1.38-6.73)	3.75** (1.53-9.20)	5.81** (1.73-19.52)	1.95* (1.14-3.31)	2.15* (1.12-4.12)	2.74** (1.36-5.50)

*p < .05, **p < .01; Interaction of glucose group and ethnicity: *p* = .0647 for any CAC and *p* = .0239 for clinically significant CAC ≥ 100. DM < 10 versus ≥10 years interaction *p* = .05 for CAC ≥ 100 only. Abbreviations: CAC = coronary artery calcium. Relevant risk factors adjusted for include age, sex, family history, education (attainment of bachelor’s degree or higher), smoking status, systolic blood pressure, diastolic blood pressure, low-density lipoprotein cholesterol, high-density lipoprotein cholesterol, triglyceride level, eGFR, waist circumference, activity level, use of lipid lowering medication and use of antihypertensive medication with *p* < .05 considered to be significant.

**Figure 2.**
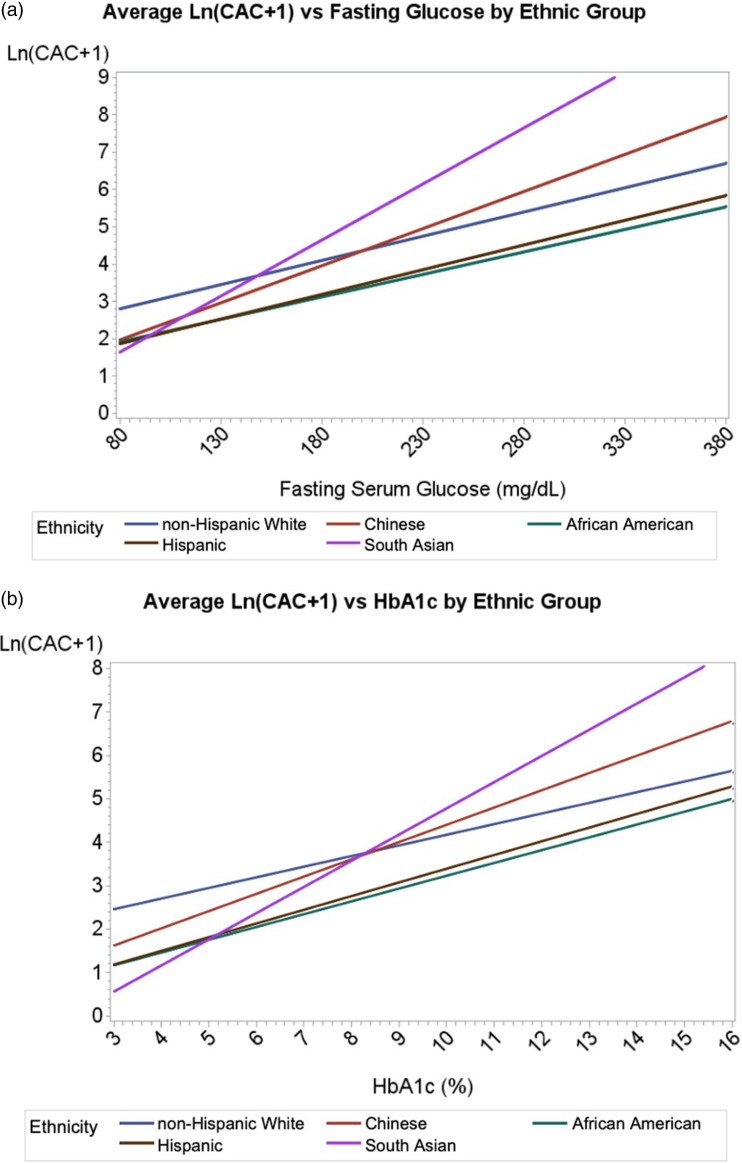
Fasting glucose versus log normalized coronary artery calcium burden by race/ethnic group. Average values of the natural log of CAC score + 1 were plotted against (a) fasting serum glucose in mg/dL (divide by 18 for mmol/L) and (b) HbA1c (%) for individuals with any CAC > 0 (*n* = 3708). Data are unadjusted for covariates. Abbreviations: CAC = coronary artery calcium, HbA1c = hemoglobin A1c, dL = deciliter, mg = milligrams.

The slope of fasting glucose (mg/dL) versus natural log-transformed CAC scores among those with CAC > 0 from multiple linear regression differed significantly between race/ethnic groups, with the steepest slopes seen for SA followed by CH adults (Figure 2(a)). We show that above fasting plasma glucose levels of approximately 150 mg/dL, the average CAC in SA adults exceeded all other groups. CH adults and NHW adults demonstrated the second and third greatest slopes of CAC versus fasting glucose, respectively, while HS adults and AA adults had the lowest. Analyses stratified by sex showed similar findings (results not shown). Moreover, the interaction of fasting glucose and race/ethnicity in predicting average CAC was significant for SA adults compared to HS or AA adults (*p* < .05 for both), but similar between SA adults and NHW adults (*p* = .49) or CH adults (*p* = .64). A similar plot was done for HbA1c (%) (Figure 2(b)). SA adults had the steepest slope followed by CH adults and NHW adults, while HS adults and AA adults had the lowest. Moreover, the interaction of HbA1c and race/ethnicity in predicting average CAC was significant for SA adults compared to HS adults (*p* = .04) or AA (*p* = .05), but similar between SA adults and NHW adults (*p* = .68) or CH adults (*p* = .90).

## Discussion

Our study including participants from the MESA and MASALA studies showed that while the overall CAC burden in SA adults was comparable to the NHW population, the presence of DM was associated with a greater likelihood of having any or clinically significant CAC in SA adults while pre-DM was not. CH adults with DM also had higher odds of clinically significant CAC, particularly in those with longer duration of DM (≥10 years). Furthermore, both higher fasting glucose and HbA1c were associated with higher CAC burden in SA and CH adults that exceeded that of other race/ethnic groups. There was no observed association between pre-DM and CAC among all subgroups. These observations suggest that DM is more strongly associated with subclinical atherosclerosis in SA and CH adults compared to NHW, HS, and AA adults.

Reports from MESA and MASALA have previously shown that SA men have similar CAC burden as NHW men but higher than other race/ethnic groups which may contribute to higher rates of cardiovascular disease in SA.^4^ A study evaluating 80 SA and white European men with known CAD using multislice CT angiography demonstrated equivalent levels of CAC but a higher prevalence of proximal left anterior descending stenosis in SA men.^12^ Another study of 1426 participants including Asian Indian, HS, AA and East Asian participants matched 3:1 with NHW participants of similar cardiovascular disease risk who underwent CAC measurement also showed similar calcium burden between NHW persons and Asian Indian individuals below the age of 60.^13^ However, in studies specifically comparing the population of SA to NHW adults with DM, SA individuals exhibited a higher calcium burden. Roos et al demonstrated in a study of 120 asymptomatic SA adults with type 2 DM who were matched with age-adjusted NHW counterparts that SA individuals had both significantly higher CAC scores and higher prevalence of significant CAD (41% vs 28%, *p* = .008).^5^ Another study of 159 SA persons with type 2 DM matched 1:1 with NHW counterparts with type 2 DM and similar cardiovascular risk profiles showed that in the DM population, SA persons had more frequent CAC and more severe CAD on coronary CT angiography compared to NHW persons.^14^

The results of this present study are consistent and show higher odds of any or clinically significant CAC in SA with DM compared to other groups, suggesting a synergistic role of DM in the pathogenesis of CAC that may be more pronounced in the SA and CH adults. This may be contributing to the subclinical atherosclerotic disease underlying the severe CAD that has been observed. In a 16-year follow-up of the Southall and Brent studies in the United Kingdom, nearly half of SA men who died from coronary events had DM compared to only 13% in European men, and having DM was associated with a nearly threefold higher risk of mortality for SA individuals.^3,15^ It has also been shown that the average CAC score for those with DM was significantly higher than those without DM (281 ± 567 vs 119 ± 341). Moreover, individuals with DM but with no CAC exhibit similar survival to those with neither DM or CAC.^16^ Although metabolic risk factors alone have not been demonstrated to account fully for the increase in CAD risk in SA adults,^13^ the higher morbidity and mortality of CAD is likely driven significantly by CAC which is disproportionately enhanced by comorbid DM compared to other ethnicities.

The greater odds of CAC we find associated with DM in SA and CH adults compared to other groups may be partly attributable to the heterogeneous spectrum of DM severity in these groups. We showed that the prevalence of any CAC is greater in SA with pre-DM and DM compared to with neither condition. These findings suggest a threshold of glucose intolerance above which CAC is of greater extent; notably above fasting serum glucose levels of 150 mg/dL, where the average CAC in SA exceeded that of other race/ethnic groups. This suggests that hyperglycemia may disproportionately affect development of CAC in SA. Indeed, SA are more likely than other race/ethnic groups to exhibit the “severe hyperglycemia” subtype of DM according to a recent study of diabetes types/subgroups among MESA and MASALA participants.^17^ Glycemic control may be especially beneficial from a preventive standpoint in SA adults with DM.

Interestingly, previous studies have shown that while SA adults were comparable to NHW adults in prevalent and incident CAC after adjusting for common comorbidities including DM, CAC in the CH population is noted to be less prevalent.^18^ Results from our study suggested that DM may accentuate the prevalence of CAC in CH adults who demonstrated higher odds of having clinically significant CAC in persons with DM compared to those without when compared to NHW adults. Furthermore, higher fasting glucose was associated with greater CAC burden in CH adults. A study of 588 participants with CAD evaluating coronary plaque and CAC scores of individuals with DM compared to without DM using dual-source CT confirmed that, in addition to having higher CAC scores and distributions in the major coronary arteries, the rate of major adverse cardiac events was also higher in CH with DM.^19^ This important finding highlights that the previously observed racial or ethnic differences in CAC observed in more general populations may not be the case in those with DM where some race/ethnic groups may be disproportionately affected by the metabolic effects of DM on CAC.

There is growing evidence of metabolic differences in SA adults compared to other race or ethnic groups that may explain the role of DM on prevalent CAC seen in this study. Differences in mitochondrial coupling efficiency which plays a role in conversion of lipid stores to adenosine triphosphate may place SA adults at higher risk of metabolic syndrome when consuming higher calorie diets in developed countries.^20^ Differences in brown adipose tissue composition in SA adults compared to NHW adults may also play a role.^21^ Prior studies using MESA and MASALA have shown that there are unfavorable differences in intramuscular fat and visceral fat with lower adiponectin and higher resistin levels in SA adults compared to NHW, CH, AA and HS adults.^22^ The duration of having DM or pre-DM may also play a role in developing any or significant CAC. A previous study of 175 individuals categorized by duration of DM who underwent coronary angiography demonstrated that having DM for 5–10 years was associated with significant coronary structural changes, while those with DM for less than 5 years were comparable to normoglycemic individuals.^23^ The higher prevalence of lean BMI DM and earlier average age of DM onset corroborates the trends observed in our study.^24^ While race and ethnicity are social constructs these variables capture important epidemiologic information, including social determinants of health, socioeconomic position, and environmental exposures. Although we do not have information on genetic ancestry, the present study uses race and ethnicity as a correlate of both biological and social variation of population groups. This approach allows for a more holistic evaluation of individual risk and may be helpful for clinical management decisions.^25^

There are several limitations to this study. Importantly, we cannot necessarily generalize our results beyond SA and CH adults living in the US, and even generalizability within the US is limited given most SA and CH participants were from California and Illinois. Differences in acculturation among our participants in the US compared to those residing outside the US may also result in differences in risk factors and/or CAC prevalence/extent. Also, while baseline CAC measures were done approximately 10 years later in MASALA compared to MESA, the scanning methodology and reading center were the same. Different ages across race/ethnic groups (with SA being the youngest, potentially attenuating effects) are also a limitation of our analysis, however age was adjusted for in the multivariate analyses. For the purposes of our study, DM was defined as a binary value which fails to capture the severity of impaired glucose metabolism or the duration with which participants have had DM, both of which are likely to influence individual risk and collective risk as an ethnic group. However, our results correlating the extent of CAC with fasting glucose across our race/ethnic groups corroborates our findings. Previous work has shown that differences in CAC trends between ethnic groups may depend on sex,^4,18^ and while our study adjusted for this covariate, a separate analysis comparing males to females with diabetes is not included as sample sizes were too small to draw meaningful conclusions from sex stratified secondary analysis. Multiple testing across DM measures and race/ethnic groups is a potential mechanism for the observed statistical significance. Recently, CAC Agatston score alone was found to not fully capture the degree of calcification effects on coronary plaque, and that CAC density and volume may also play a role.^26^ This study was not designed to evaluate the effect of DM on those parameters which are gaining significance in prognostication. Moreover, our studies did not have more novel measures of adiposity, such as brown adipose tissue, and future studies should consider such measurements.

In conclusion, DM was associated with highest likelihood of any CAC in SA and NHW adults and clinically significant CAC in SA and CH adults. Higher values of fasting glucose were associated with greater CAC burden in SA adults than NHW adults and other race/ethnic groups. The interaction of DM and CAC may play a significant role in the higher morbidity and mortality associated with ASCVD in the SA and CH population. These findings suggest poor glycemic control may impact the development of atherosclerosis more in these Asian American groups than in others, further emphasizing the role of optimal glycemic control in these groups from a preventive standpoint. Prospective studies evaluating the relative benefits of glycemic control in severity of CAD between race/ethnic subgroups may be useful for further insight.
